# Use of a Machine Learning Program to Correctly Triage Incoming Text Messaging Replies From a Cardiovascular Text–Based Secondary Prevention Program: Feasibility Study

**DOI:** 10.2196/19200

**Published:** 2020-06-16

**Authors:** Nicole Lowres, Andrew Duckworth, Julie Redfern, Aravinda Thiagalingam, Clara K Chow

**Affiliations:** 1 Heart Research Institute Sydney Australia; 2 Faculty of Medicine and Health University of Sydney Sydney Australia; 3 Westmead Applied Research Centre University of Sydney Sydney Australia; 4 Department of Cardiology Westmead Hospital Sydney Australia

**Keywords:** eHealth, machine learning, secondary prevention, SMS text messaging, cardiovascular, mHealth, digital health, mobile phone

## Abstract

**Background:**

SMS text messaging programs are increasingly being used for secondary prevention, and have been shown to be effective in a number of health conditions including cardiovascular disease. SMS text messaging programs have the potential to increase the reach of an intervention, at a reduced cost, to larger numbers of people who may not access traditional programs. However, patients regularly reply to the SMS text messages, leading to additional staffing requirements to monitor and moderate the patients’ SMS text messaging replies. This additional staff requirement directly impacts the cost-effectiveness and scalability of SMS text messaging interventions.

**Objective:**

This study aimed to test the feasibility and accuracy of developing a machine learning (ML) program to triage SMS text messaging replies (ie, identify which SMS text messaging replies require a health professional review).

**Methods:**

SMS text messaging replies received from 2 clinical trials were manually coded (1) into “Is staff review required?” (binary response of yes/no); and then (2) into 12 general categories. Five ML models (Naïve Bayes, OneVsRest, Random Forest Decision Trees, Gradient Boosted Trees, and Multilayer Perceptron) and an ensemble model were tested. For each model run, data were randomly allocated into training set (2183/3118, 70.01%) and test set (935/3118, 29.98%). Accuracy for the yes/no classification was calculated using area under the receiver operating characteristics curve (AUC), false positives, and false negatives. Accuracy for classification into 12 categories was compared using multiclass classification evaluators.

**Results:**

A manual review of 3118 SMS text messaging replies showed that 22.00% (686/3118) required staff review. For determining need for staff review, the Multilayer Perceptron model had highest accuracy (AUC 0.86; 4.85% false negatives; and 4.63% false positives); with addition of heuristics (specified keywords) fewer false negatives were identified (3.19%), with small increase in false positives (7.66%) and AUC 0.79. Application of this model would result in 26.7% of SMS text messaging replies requiring review (true + false positives). The ensemble model produced the lowest false negatives (1.43%) at the expense of higher false positives (16.19%). OneVsRest was the most accurate (72.3%) for the 12-category classification.

**Conclusions:**

The ML program has high sensitivity for identifying the SMS text messaging replies requiring staff input; however, future research is required to validate the models against larger data sets. Incorporation of an ML program to review SMS text messaging replies could significantly reduce staff workload, as staff would not have to review all incoming SMS text messages. This could lead to substantial improvements in cost-effectiveness, scalability, and capacity of SMS text messaging–based interventions.

## Introduction

Cardiovascular disease (CVD) is the leading cause of death globally [1]. Secondary prevention interventions, such as cardiac rehabilitation, are very effective at reducing CVD and preventing cardiovascular events [2,3]. However, access to traditional cardiac rehabilitation and secondary prevention is limited due to many well-described barriers [4,5]. As a result, alternative methods of secondary prevention, including electronic health (eHealth) and mobile health (mHealth), are emerging with demonstrated effectiveness [6]. Because 88% of Australians (aged 18-75 years) now own a smartphone [7], the potential reach of mHealth programs to deliver CVD secondary prevention is significant. Smartphone mHealth interventions are shown to be an acceptable and feasible method of health care delivery [8,9]. Specifically, SMS text messaging programs have been shown to be effective for cardiac risk factor reduction [10-12], medication adherence [13,14], weight loss [15], and physical activity [16,17]. However, one study showed limited maintenance of behavior changes once the SMS text messaging intervention ceased [17]. SMS text messaging programs have also been utilized effectively in many other medical settings including diabetes [18], maternal health [19], hepatitis B [20], and smoking cessation [21].

SMS text messaging programs can be set up as either a one- or a two-way messaging communication system. The TEXTME program was an automated semipersonalized SMS text messaging program to support lifestyle change for people with coronary heart disease, which was originally delivered as a one-way communication system [12]. TEXTME sent participants 4 SMS text messages per week for 6 months, and demonstrated significant improvements in cholesterol, blood pressure, BMI, physical activity, and smoking reduction, compared with usual care [12]. In addition, participants were highly satisfied and engaged with the program, reporting numerous additional psychosocial benefits [12,22]. However, even in this one-way SMS text messaging program, where participants were specifically instructed not to respond, 32.9% (116/352) replied to the SMS text messages, with many replying multiple times [22]. The cost of delivering automated SMS text messages is relatively inexpensive (approximately AUD 13/person [US $8.60/person] for the entire program) [12]. However, when participants respond to SMS text messages there are various staffing and logistical requirements and a trained health professional is required to moderate replies. In TEXTME many replies were statements of thanks, but sometimes SMS text messages require a professional response, particularly in the case of medical distress [22], or if participants wish to withdraw from the program.

Machine learning (ML) programs have commonly been used in health care to predict outcomes [23,24], but in other applications they have been used to classify text [25,26]. It is therefore possible that a computerized ML program could be developed and trained to accurately identify which incoming SMS text messages require review by a health professional. If successful, an ML program could significantly reduce the health professional’s workload. Thus, the cost of running an SMS text messaging–based intervention would be significantly reduced, and the scalability and capacity of the program would improve substantially. Therefore, this study aimed to test the feasibility of developing an ML program to triage and identify SMS text messages requiring a health professional’s review. Specifically, the primary aim was to determine the accuracy of the ML program to sort which SMS text messaging replies require a staff member review (ie, binary classification of yes/no); and the secondary aim was to assess the accuracy of the ML program to classify each SMS text messaging reply into overall categories or themes (ie, multiclass classification).

## Methods

### Study Population

The SMS text messaging replies used in this analysis originated from the TEXTME and TEXTMEDS programs [12,27]. TEXTME was a 6-month program of SMS text messages for patients with coronary heart disease, where patients were explicitly instructed not to respond to SMS text messages (ACTRN12611000161921) [12]. TEXTMEDS was a 12-month program for patients with acute coronary syndrome, with a more interactive two-way SMS text messaging protocol (ACTRN12613000793718) [27]. Both studies recruited patients during hospitalization. The demographics of the TEXTME patients have previously been published [12], which included the following: mean age 57.9 (SD 9.1) years; 81.5% (287/352) men; and education level of 11 years (interquartile range 9-13).

### Manual Coding of SMS Text Messaging Replies

In this study, de-identified SMS text messaging replies from the TEXTME and TEXTMEDS studies were extracted into a database. SMS text messages were entered into the database exactly as they were received, with no correction to spelling, grammar, or punctuation. The TEXTME and TEXTMEDS programs had created 22 categories of SMS text messaging replies for administrative and management purposes (eg, according to content, administrative request, or general commentary; Multimedia Appendix 1).

Two experienced cardiac health professionals (NL and Anu Indrawansa, Westmead Hospital, Sydney, Australia) independently reviewed and coded each SMS text message into the 22 categories according to the theme of the message. For the SMS text messages that expressed more than 1 theme, categorization was done according to the main theme or the theme that required a staff member review/action. For SMS text messages where a consensus on coding could not be reached, a third person (AD) was consulted. Using an iterative process, these 22 categories were condensed by NL and AD into 12 categories (Table 1), with consideration given to the type of response required for the SMS text message. Finally, each SMS text message was designated into one of two groups, depending on whether it required a staff member review or not (ie, yes or no).

### Machine Learning Model Development

Five different ML models and an ensemble model were tested using Apache Spark (version 2.3.4) and the models, and their associated parameters or weights or both, were saved for further analysis. First, we assessed 4 models which are considered good models for classification purposes: Naïve Bayes, OneVsRest, Random Forest decision trees, and Gradient Boosted Trees. These models use a combination of different statistical data analytics and regression tools to assess the data and determine the likelihood of the correct response. Second, we assessed a convolutional neural network classification approach: Multilayer Perceptron. Neural networks use layers of iterative feedback cycles to calculate the outcome, and are commonly used for natural language translation in systems such as Google Translate, and for speech and image recognition. Lastly, we implemented a technique that combined the results from different models together as an *Ensemble model*. We evaluated the effect of using heuristics (ie, selected keywords) in each model, by programming and testing each model with and without the inclusion of heuristics. We also evaluated the addition of *natural language programming* which interprets and determines the intended context of written statements.

**Figure 1:**
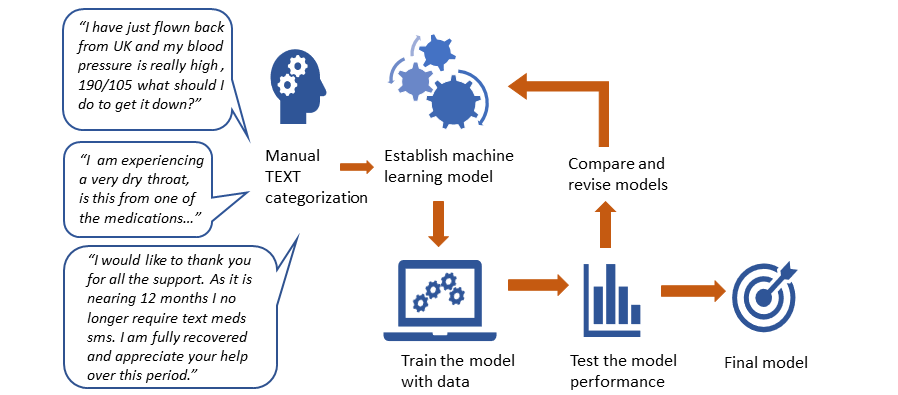
Machine learning model development and testing.

Each time a model was run, the data set was randomly divided into a training set (2183/3118, 70.01%) and a test set (935/3118, 29.98%), thus creating a unique allocation of SMS text messages to the training and test data each time. Each model was assessed and compared for determining if health professional review was required (ie, binary variable of yes/no; Figure 1). In addition, we assessed and compared each model that supported multiclass classification (Gradient Boosted Trees only supports binary classification) for its ability to correctly classify the SMS text messages into the 12 categorical variables as listed in Table 1. Each model was run 5 times on different random splits of the training and test data sets to validate the accuracy of the results.

**Table 1:** Manual classification of SMS text messaging replies (N=3118)

Classification category	Health professional review required	
	Yes^a^, n (%)	No^b^, n (%)
Health question/concern	383 (12.28)	–
Administrative request	155 (4.97)	–
Request to STOP/pause	107 (3.43)	–
Ceased smoking	24 (0.76)	–
SMS text message not delivered	14 (0.44)	–
Timely response required	4 (0.12)	–
General statement	–	1132 (36.30)
Statement of thanks	–	789 (25.30)
Reporting good health	–	216 (6.92)
Blank SMS text message	–	191 (6.12)
Positive emoticon	–	74 (2.37)
Unrelated/accidental	–	29 (0.93)

^a^Total: 687/3118 (22.03%).

^b^Total: 2431/3118 (77.96%).

### Statistical Analysis and Comparison of Models

For the primary outcome of determining which SMS text messaging replies require a staff member review (binary outcome of yes/no), a binary classification evaluator was used to calculate accuracy using the area under the receiver operating characteristics curve (AUC), the rate of true and false positives, and the rate of true and false negatives. For the secondary outcome of the accuracy of the ML program to classify the SMS text messaging replies into the 12 categories, a multiclass classification evaluator was used to compare each model for accuracy, precision, recall, and F1 score.

## Results

### Manual Classification of SMS Text Messaging Replies

A manual review of 3118 SMS text messaging replies was performed. The manual classification identified 12 broad categories of replies (Table 1). Only 22.03% (687/3118) of all SMS text messages required someone to review and reply to the SMS text messages, and only 0.12% (4/3118) SMS text messages were deemed to warrant a timely response within 24-48 h; however no SMS text messages were considered urgent (Table 1).

### Machine Learning Model Development

Natural language programming was evaluated as an option to improve the ML model’s ability to understand the sentiment behind each SMS text message. Ultimately, natural language programming was not included in any of the models as challenges arose in relation to the SMS text messaging content, due to the frequent use of nonstandard English grammar, nonstandard abbreviations, and spelling errors within the SMS text messages.

#### Accuracy for Determining If Staff Review Is Required

**Figure 2:**
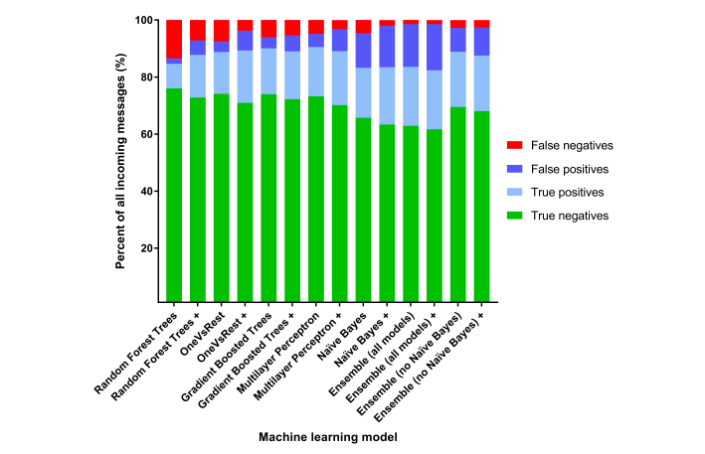
Machine learning model performance. +: model with heuristics added.

The results for each model, with and without the inclusion of heuristics, are outlined in Figure 2 and Multimedia Appendix 2. The inclusion of heuristics reduced the number of false negatives in each of the models, at the expense of an increase in the number of false positives. From the individual models, Naïve Bayes with heuristics produced the lowest false negatives (1.98%) and the highest false positives (14.54%). The Random Forest decision trees model resulted in the lowest number of false positives (1.87%), but had the highest rate of false negatives (13.44%).

**Figure 3:**
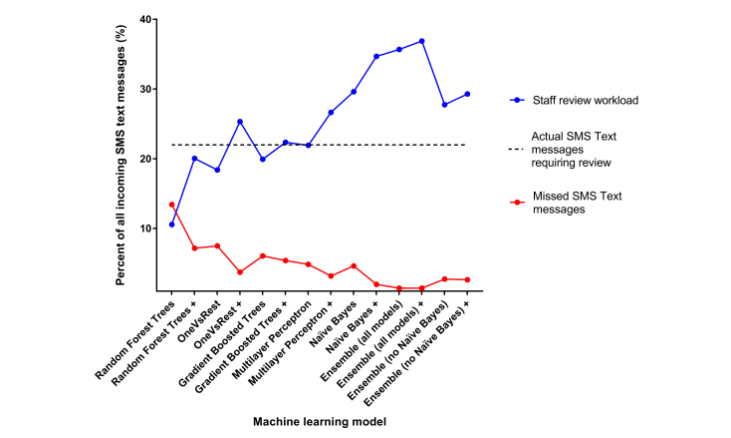
Staff workload versus missed messages according to machine learning model. Staff review workload: true positives plus false positives; +: model with heuristics added.

The Multilayer Perceptron model correctly identified if staff review was required (yes/no) in 90.52% of cases (4.85% false negatives and 4.63% false positives from the test data sample). With addition of heuristics to the Multilayer Perceptron model, fewer false negatives were identified (3.19%) with a small increase in false positives (7.71%). Utilizing the Multilayer Perceptron model with heuristics, staff would have to review only 26.65% of all SMS text messaging replies (ie, 18.94% true positives + 7.71% false positives; Figure 3).

However, with the aim of reducing the false negatives to the lowest possible number, the ensemble model was found to be superior (1.43% false negatives and 16.2% false positives), giving a sensitivity of 93.5% for identifying the SMS text messages requiring review, and a specificity of 81.3%. Using the ensemble model with heuristics, the results indicated that health professionals would have to review 36.9% (ie, 20.7% true positives + 16.2% false positives) of all incoming SMS text messaging replies (Figure 3).

Examples of SMS text messages that were incorrectly coded by the Multilayer Perceptron model as “Yes – review required”:

I am now careful with my choices when i purches meats etc bacon is not eaten very often like it used to be

Pill are not a problem for me. To this point limited to no side effects. Just got to keep up with exercise regime.

Examples of SMS text messages that were incorrectly coded by the Multilayer Perceptron model as “No review required”:

Hi everything is excellent. Eating well exercising everyday taking medicine. You could probably take me of list as everything you suggest has already been implemented from day one of recovery. Thanks for your input. Regards

Just a query , I walk for 1/2 each day , is this sufficient?

#### Accuracy to Identify the Priority SMS Text Messages

Importantly, the model correctly identified the 4 priority SMS text messages that required a timely review. The model missed only 1 of 107 SMS text messages where the participant was requesting to opt out of the program or pause the SMS text messages. The only SMS text message that was missed read “no more texts now thanks.”

#### Accuracy for Classification Into 12 Categories

For classification into the 12 categories, OneVsRest and the Multilayer Perceptron models produced the best results. OneVsRest had slightly higher accuracy of 0.723, with precision 0.723, recall 0.723, and F1 score 0.719. For the Multilayer Perceptron model, accuracy was 0.717, precision 0.735, recall 0.717, and F1 score 0.723.

## Discussion

### Principal Findings

Our study demonstrates the feasibility and accuracy of using an ML program as a triage system to sort which incoming SMS text messaging replies require health professional review, in a cardiovascular secondary prevention SMS text messaging intervention setting. Each of the ML models tested varied in their sensitivity and specificity for classifying the SMS text messages, and there appeared to be a trade-off between false negatives and false positives. From the individual models, Naïve Bayes produced the lowest false-negative rate, and Random Forest decision trees produced the lowest false-positive rate. In our study, the ensemble model (with all models combined) was the most accurate at identifying the SMS text messages needing review, with only 1.43% of the SMS text messages being false negatives. If this ensemble model were utilized as a triage for incoming SMS text messages, health professionals would have to review about 37% of all the incoming SMS text messaging replies. For sorting the SMS text messages into 12 categories (according to the theme of the SMS text message), the accuracy was only moderate (0.723), indicating further work is needed to create suitable categories and to train the model with larger data sets and examples.

To our knowledge this is the first study in a health setting to use an ML program to classify (ie, triage) incoming SMS text messages. ML has been widely validated and used for the purpose of filtering SPAM SMS text messages, classifying SMS text messages into a binary outcome (SPAM or not SPAM) which is similar to our study [28,29]. These studies on SPAM SMS text messages have employed different approaches for reviewing content, filtering, and feature extraction; however, they have produced similar accuracy results to our study [28,29]. The use of ML SPAM filters within the operating systems of smartphones is now commonplace, with early adoption dating back to Apple’s iOS 11 in 2017.

Within the health industry, ML algorithms are common in technology such as electrocardiogram analysis; however, the use of ML to interpret medical text or within patient–provider interactions has not been adopted in everyday practice. There may be larger ethical barriers to overcome in the adoption of ML programs if they are used to assist health care decisions and advice, due to the duty of care to our patients and concern over the consequences if the ML program misses a critical medical alert. Research using ML in the health setting has predominantly focused on extracting information from fields in health and medical records to predict the risk of an outcome such as mortality [30,31], emergency re-admissions [32], and myopia development [33]. It is difficult to compare accuracy across these studies as they measured different outcomes and reported accuracy using different metrics, however they generally performed well, with accuracy examples of AUC > 0.82 for predicting mortality after echocardiography [30]; and AUC range of 0.87-0.98 for predicting myopia development [33]. There is one study which has used ML as a *triage* for patients with congenital heart disease to estimate the need for a multidisciplinary review [34]. With all these studies, the ML models were trained to review fields in the medical records to determine the likelihood of the outcome, and therefore did not have to interpret the meaning or sentiment of the text in the medical notes [34].

More recently, ML has successfully been applied to more unstructured written health notes and dialogues [35,36]. One study was able to phenotype depression using unstructured notes in the medical records with a sensitivity of 93.5% and specificity of 68% [35]. Natural language processing has also been used to review secure online discussions between patients and health professionals, in which linguistic features were assessed to predict health literacy levels with an accuracy of 60.55% (C-statistic 0.63) [36]. Common to these studies, there was one ML model which performed better than the other models, and the model varied across the studies dependent on the primary data and outcome they were measuring, and this is also reflected in our results.

### Clinical Implications

Although ML research in health care is still evolving, the possible breadth of using ML is promising. The most valuable feature of the ML program in our study was the ability to identify the SMS text messages that were needed to be reviewed by a staff member. Therefore, we were interested in identifying the model with the lowest false-negative rate. The AUC was not the best statistic for assessing the model performance, as for each small decrease in false negatives, there was a much larger increase in false positives, and thus a reduction in AUC.

If the ensemble model, with the lowest false negatives, was implemented in a secondary prevention SMS text messaging program, the minimal number of SMS text messages will be missed, but this would be at the expense of staff needing to review more overall SMS text messages (due to the higher false positives). However, even with reviewing additional false positives, staff would only need to review approximately 37% of all incoming SMS text messages, which will significantly reduce the workload of the health professionals running the program, permitting more efficient use of their time. Although it is thought that SMS text messaging interventions may be cost-effective [37], the cost-effectiveness of this solution would need to be evaluated prior to implementation.

Furthermore, prior to implementation it is also important to ensure that the model is capturing all of the most important SMS text messages requiring action from the health professional, and that there is no systemic misclassification. This would need to be tested with larger databases, with careful monitoring of the high-risk categories, to ensure the model is doing what we want it to do at scale. The use of heuristics in the model provides an additional *safeguard* to ensure that SMS text messages with certain keywords are reviewed. If participants are provided with specific words to opt out of SMS text messaging programs, such as STOP, then heuristics are even more effective at identifying all participants trying to opt out of the program.

Once implemented, the ML program would run alongside the SMS text messaging program, and would continue to be trained as the database grows, thereby improving the accuracy of the ML algorithms. To further improve the certainty of SMS text message classification, it would be possible to program BOT-like features into the model to automatically reply to the sender if clarification of the need to respond is required (eg, “Hi...Would you like me to contact you to discuss this?”). Furthermore, if the accuracy of the ML model could be improved for the classification of SMS text message into more specific categories, it may be possible to consider developing an automated system that sends an appropriate motivational response for the SMS text messages that do not require a staff member response.

### Strengths and Limitations

This is the first study in a health setting to explore the use of ML for classification of health-related SMS text messages. It assesses and compares the accuracy of 7 different ML models, and employs a robust method for verification of results using 5 different splits of the training and test data sets. In all triage systems that require a human to decide on the priority and need for medical care, there is a certain element of subjective bias in the decision-making process. In our study where health professionals classified the incoming SMS text messages into whether or not a review was required, an element of subjectivity in that decision remained. This decision making is also complicated by many of the SMS text messages expressing one or more sentiments, and a subjective decision being made as to the most important sentiment to classify the SMS text messages. As there is no gold standard to define whether an SMS text message needs to be reviewed or not, it is possible that health professionals with different areas or levels of experience may manually classify the incoming SMS text messages differently. Our study demonstrates a proof-of-concept, as we tested a limited number of SMS text messaging replies (n=3118); however, to generalize the results beyond our study population, future work is required to validate the model against larger data sets, and on data sets that are coded by different health professionals.

### Conclusions

In our feasibility study, the ML program displayed high sensitivity in identifying the SMS text messaging replies that require health professional input. There is a low false-negative rate, indicating few messages which need a response would be missed. Introduction of the ML program to SMS text messaging–based programs could therefore significantly reduce the need for health professionals to review every incoming SMS text message. This could lead to substantial improvements in scalability and capacity of SMS text messaging–based programs. The future implications for this technology are vast, including potential utilization in other interactive mHealth interfaces and cardiovascular health apps.

## Appendix

Multimedia Appendix 1 Original Categorisation used in TEXTME and TEXTMEDS study.

Multimedia Appendix 2 Results for identification of texts requiring review.

## Abbreviations

AUC: area under the receiver operating characteristics curve
CVD: cardiovascular disease
ML: machine learning

## References

[1] World Health Organisation (2017). Cardiovascular Disease Fact Sheet 2017.

[2] Clark AM, Hartling L, Vandermeer B, Lissel SL, McAlister FA (2007). Secondary prevention programmes for coronary heart disease: a meta-regression showing the merits of shorter, generalist, primary care-based interventions. Eur J Cardiovasc Prev Rehabil.

[3] Clark AM, Hartling L, Vandermeer B, McAlister FA (2005). Meta-analysis: secondary prevention programs for patients with coronary artery disease. Ann Intern Med.

[4] Beswick AD, Rees K, Griebsch I, Taylor FC, Burke M, West RR, Victory J, Brown J, Taylor RS, Ebrahim S (2004). Provision, uptake and cost of cardiac rehabilitation programmes: improving services to under-represented groups. Health Technol Assess.

[5] Suaya JA, Shepard DS, Normand ST, Ades PA, Prottas J, Stason WB (2007). Use of cardiac rehabilitation by Medicare beneficiaries after myocardial infarction or coronary bypass surgery. Circulation.

[6] Park LG, Beatty A, Stafford Z, Whooley MA (2016). Mobile phone interventions for the secondary prevention of cardiovascular disease. Prog Cardiovasc Dis.

[7] Deloitte (2017). 2017 Mobile Consumer Survery - Australia.

[8] Payne HE, Lister C, West JH, Bernhardt JM (2015). Behavioral functionality of mobile apps in health interventions: a systematic review of the literature. JMIR Mhealth Uhealth.

[9] Badawy SM, Kuhns LM (2016). Economic evaluation of text-messaging and smartphone-based interventions to improve medication adherence in adolescents with chronic health conditions: a systematic review. JMIR Mhealth Uhealth.

[10] Adler AJ, Martin N, Mariani J, Tajer CD, Owolabi OO, Free C, Serrano NC, Casas JP, Perel P (2017). Mobile phone text messaging to improve medication adherence in secondary prevention of cardiovascular disease. Cochrane Database Syst Rev.

[11] Thakkar J, Redfern J, Thiagalingam A, Chow CK (2016). Patterns, predictors and effects of texting intervention on physical activity in CHD - insights from the TEXT ME randomized clinical trial. Eur J Prev Cardiol.

[12] Chow CK, Redfern J, Hillis GS, Thakkar J, Santo K, Hackett ML, Jan S, Graves N, de KL, Barry T, Bompoint S, Stepien S, Whittaker R, Rodgers A, Thiagalingam A (2015). Effect of lifestyle-focused text messaging on risk factor modification in patients with coronary heart disease: a randomized clinical trial. JAMA.

[13] Thakkar J, Kurup R, Laba T, Santo K, Thiagalingam A, Rodgers A, Woodward M, Redfern J, Chow CK (2016). Mobile telephone text messaging for medication adherence in chronic disease: a meta-analysis. JAMA Intern Med.

[14] Huang H, Li YJ, Chou Y, Hsieh Y, Kuo F, Tsai W, Chai SD, Lin BY, Kung P, Chuang C (2013). Effects of and satisfaction with short message service reminders for patient medication adherence: a randomized controlled study. BMC Med Inform Decis Mak.

[15] Patrick K, Raab F, Adams MA, Dillon L, Zabinski M, Rock CL, Griswold WG, Norman GJ (2009). A text message-based intervention for weight loss: randomized controlled trial. J Med Internet Res.

[16] Martin SS, Feldman DI, Blumenthal RS, Jones SR, Post WS, McKibben RA, Michos ED, Ndumele CE, Ratchford EV, Coresh J, Blaha MJ (2015). mActive: a randomized clinical trial of an automated mHealth intervention for physical activity promotion. J Am Heart Assoc.

[17] Müller AM, Khoo S, Morris T (2016). Text messaging for exercise promotion in older adults from an upper-middle-income country: randomized controlled trial. J Med Internet Res.

[18] Dobson R, Carter K, Cutfield R, Hulme A, Hulme R, McNamara C, Maddison R, Murphy R, Shepherd M, Strydom J, Whittaker R (2015). Diabetes text-message self-management support program (SMS4BG): a pilot study. JMIR Mhealth Uhealth.

[19] Dobson R, Whittaker R, Bartley H, Connor A, Chen R, Ross M, McCool J (2017). Development of a culturally tailored text message maternal health program: TextMATCH. JMIR Mhealth Uhealth.

[20] Hyun C, McMenamin J, Ko O, Kim S (2020). Efficacy of a mobile texting app (HepTalk) in encouraging patient participation in viral hepatitis B care: development and cohort study. JMIR Mhealth Uhealth.

[21] Gram IT, Larbi D, Wangberg SC (2019). Comparing the efficacy of an identical, tailored smoking cessation intervention delivered by mobile text messaging versus email: randomized controlled trial. JMIR Mhealth Uhealth.

[22] Redfern J, Santo K, Coorey G, Thakkar J, Hackett M, Thiagalingam A, Chow CK (2016). Factors influencing engagement, perceived usefulness and behavioral mechanisms associated with a text message support program. PLoS One.

[23] Zack CJ, Senecal C, Kinar Y, Metzger Y, Bar-Sinai Y, Widmer RJ, Lennon R, Singh M, Bell MR, Lerman A, Gulati R (2019). Leveraging machine learning techniques to forecast patient prognosis after percutaneous coronary intervention. JACC Cardiovasc Interv.

[24] Al'Aref SJ, Anchouche K, Singh G, Slomka PJ, Kolli KK, Kumar A, Pandey M, Maliakal G, van Rosendael AR, Beecy AN, Berman DS, Leipsic J, Nieman K, Andreini D, Pontone G, Schoepf UJ, Shaw LJ, Chang H, Narula J, Bax JJ, Guan Y, Min JK (2019). Clinical applications of machine learning in cardiovascular disease and its relevance to cardiac imaging. Eur Heart J.

[25] Lu Y (2013). Automatic topic identification of health-related messages in online health community using text classification. Springerplus.

[26] Chatterjee S, Deng S, Liu J, Shan R, Jiao W (2018). Classifying facts and opinions in Twitter messages: a deep learning-based approach. Journal of Business Analytics.

[27] Chow CK, Thiagalingam A, Santo K, Kok C, Thakkar J, Stepien S, Billot L, Jan S, Joshi R, Hillis GS, Brieger D, Chew DP, Rådholm K, Atherton JJ, Bhindi R, Collins N, Coverdale S, Hamilton-Craig C, Kangaharan N, Maiorana A, McGrady M, Shetty P, Thompson P, Rogers A, Redfern J (2018). TEXT messages to improve MEDication adherence and Secondary prevention (TEXTMEDS) after acute coronary syndrome: a randomised clinical trial protocol. BMJ Open.

[28] Waheeb W, Ghazali R (2018). Content-based SMS classification: statistical analysis for the relationship between number of features and classification performance. Comput Syst.

[29] Choudhary N, Jain A, Singh D, Raman B, Luhach AK, Lingras P (2017). Towards filtering of SMS spam messages using machine learning based technique. Advanced Informatics for Computing Research.

[30] Samad MD, Ulloa A, Wehner GJ, Jing L, Hartzel D, Good CW, Williams BA, Haggerty CM, Fornwalt BK (2019). survival from large echocardiography and electronic health record datasets: optimization with machine learning. JACC Cardiovasc Imaging.

[31] Steele AJ, Denaxas SC, Shah AD, Hemingway H, Luscombe NM (2018). Machine learning models in electronic health records can outperform conventional survival models for predicting patient mortality in coronary artery disease. PLoS One.

[32] Rahimian F, Salimi-Khorshidi G, Payberah AH, Tran J, Ayala Solares R, Raimondi F, Nazarzadeh M, Canoy D, Rahimi K (2018). Predicting the risk of emergency admission with machine learning: development and validation using linked electronic health records. PLoS Med.

[33] Lin H, Long E, Ding X, Diao H, Chen Z, Liu R, Huang J, Cai J, Xu S, Zhang X, Wang D, Chen K, Yu T, Wu D, Zhao X, Liu Z, Wu X, Jiang Y, Yang X, Cui D, Liu W, Zheng Y, Luo L, Wang H, Chan C, Morgan IG, He M, Liu Y (2018). Prediction of myopia development among Chinese school-aged children using refraction data from electronic medical records: a retrospective, multicentre machine learning study. PLoS Med.

[34] Diller G, Kempny A, Babu-Narayan SV, Henrichs M, Brida M, Uebing A, Lammers AE, Baumgartner H, Li W, Wort SJ, Dimopoulos K, Gatzoulis MA (2019). Machine learning algorithms estimating prognosis and guiding therapy in adult congenital heart disease: data from a single tertiary centre including 10 019 patients. Eur Heart J.

[35] Geraci J, Wilansky P, de LV, Roy A, Kennedy JL, Strauss J (2017). Applying deep neural networks to unstructured text notes in electronic medical records for phenotyping youth depression. Evid Based Ment Health.

[36] Balyan R, Crossley SA, Brown W, Karter AJ, McNamara DS, Liu JY, Lyles CR, Schillinger D (2019). Using natural language processing and machine learning to classify health literacy from secure messages: The ECLIPPSE study. PLoS One.

[37] Badawy SM, Kuhns LM (2016). Economic evaluation of text-messaging and smartphone-based interventions to improve medication adherence in adolescents with chronic health conditions: a systematic review. JMIR Mhealth Uhealth.

