# Supplementary material for: Use of a Machine Learning Program to Correctly Triage Incoming Text Messaging Replies From a Cardiovascular Text–Based Secondary Prevention Program: Feasibility Study
Source: JMIR Mhealth Uhealth. 2020 Jun 16;8(6):e19200. doi: 10.2196/19200 (PMC7327598; doi:10.2196/19200)
Supplement: Multimedia Appendix 1 [file mhealth_v8i6e19200_app1.docx]

| **Appendix 1: Original Categorisation used in TEXTME and TEXTMEDS study** | |
| --- | --- |
| **Code** | **Category** |
| 1 | Thanks |
| 2 | Reporting they are healthy |
| 3 | General comment |
| 4 | Administrative comment |
| 5 | Blank message |
| 6 | Diet question |
| 7 | Blood pressure question |
| 8 | Medication question |
| 9 | Physical activity question |
| 10 | Related to Smoking or alcohol |
| 11 | General health question |
| 12 | Reporting medication adherence |
| 13 | Not study related |
| 14 | Invalid number or failed delivery |
| 15 | Request further information |
| 16 | Stop |
| 17 | Incorrect number |
| 18 | Going away |
| 19 | Too busy to continue |
| 20 | Incorrect reply of STOP |
| 21 | STOP requested but agreed to continue |
| 22 | Unicode (emoticon) |
