# Supplementary material for: Use of a Machine Learning Program to Correctly Triage Incoming Text Messaging Replies From a Cardiovascular Text–Based Secondary Prevention Program: Feasibility Study
Source: JMIR Mhealth Uhealth. 2020 Jun 16;8(6):e19200. doi: 10.2196/19200 (PMC7327598; doi:10.2196/19200)
Supplement: Multimedia Appendix 2 [file mhealth_v8i6e19200_app2.docx]

| **Appendix 2: Results for identification of texts requiring review** | | | | | |
| --- | --- | --- | --- | --- | --- |
| **Machine learning model** | **True positives^a^**  **(%)** | **False negatives^b^**  **(%)** | **True negatives^b^**  **(%)** | **False positives^a^**  **(%)** | **AUC** |
| Naïve Bayes | 17.51 | 4.63 | 65.75 | 12.11 | 0.82 |
| *+ heuristics* | 20.15 | 1.98 | 63.33 | 14.54 | 0.82 |
| OneVsRest | 14.65 | 7.49 | 74.12 | 3.74 | 0.81 |
| *+ heuristics* | 18.39 | 3.74 | 70.93 | 6.94 | 0.77 |
| Gradient boosted trees | 16.08 | 6.06 | 74.01 | 3.85 | 0.84 |
| *+ heuristics* | 16.74 | 5.40 | 72.25 | 5.62 | 0.68 |
| Random Forest decision trees | 8.70 | 13.44 | 75.99 | 1.87 | 0.68 |
| *+ heuristics* | 14.98 | 7.16 | 72.80 | 5.07 | 0.59 |
| Multi-Layer Perceptron | 17.29 | 4.85 | 73.24 | 4.63 | 0.86 |
| *+ heuristics* | 18.94 | 3.19 | 70.15 | 7.71 | 0.79 |
| Ensemble model  (including all 5 models) | 20.70 | 1.43 | 62.89 | 14.98 | - |
| *+ heuristics* | 20.70 | 1.43 | 61.67 | 16.19 | - |
| Modified ensemble model  (excluding Naïve Bayes) | 19.38 | 2.75 | 69.49 | 8.37 | - |
| *+ heuristics* | 19.49 | 2.64 | 68.06 | 9.80 | - |
| **^a^** Positives refer to texts that require a review; **^b^** Negatives refer to texts that do not require a review; AUC: area under the receiver operating characteristics curve (where 1.0 = 100% correct prediction; and 0.5 = poor prediction or random chance) | | | | | |
